# Liver cT_1_ decreases following direct-acting antiviral therapy in patients with chronic hepatitis C virus

**DOI:** 10.1007/s00261-020-02860-5

**Published:** 2020-11-28

**Authors:** Arjun N. A. Jayaswal, Christina Levick, Jane Collier, Elizabeth M. Tunnicliffe, Matthew D. Kelly, Stefan Neubauer, Eleanor Barnes, Michael Pavlides

**Affiliations:** 1 Oxford Centre for Clinical Magnetic Resonance Research, Division of Cardiovascular Medicine, Radcliffe Department of Medicine, University of Oxford, Oxford, UK; 2 Translational Gastroenterology Unit, University of Oxford, Oxford, UK; 3 Oxford NIHR Biomedical Research Centre, Oxford, UK; 4 Perspectum Ltd, Oxford, UK; 5 Peter Medawar Building for Pathogen Research, University of Oxford, Oxford, UK

**Keywords:** T_1_ mapping, Iron corrected T_1_, Direct-acting antivirals, Hepatitis C virus

## Abstract

**Purpose:**

Direct-acting antiviral therapies (DAAs) for treatment of chronic hepatitis C virus (HCV) have excellent rates of viral eradication, but their effect on regression of liver fibrosis is unclear. The primary aim was to use magnetic resonance imaging (MRI) and spectroscopy (MRS) to evaluate changes in liver fibrosis, liver fat and liver iron content (LIC) in patients with chronic HCV following treatment with DAAs.

**Methods:**

In this prospective study, 15 patients with chronic HCV due to start treatment with DAAs and with transient elastography (TE) > 8 kPa were recruited consecutively. Patients underwent MRI and MRS at baseline (before treatment), and at 24 weeks and 48 weeks after the end of treatment (EoT) for the measurement of liver cT_1_ (fibroinflammation), liver fat and T_2_* (LIC).

**Results:**

All patients achieved a sustained virological response. Liver cT_1_ showed significant decreases from baseline to 24 weeks post EoT (876 vs 806 ms, *p* = 0.002, *n* = 15), baseline to 48 weeks post EoT (876 vs 788 ms, *p* = 0.0002, *n* = 13) and 24 weeks post EoT to 48 weeks post EoT (806 vs 788 ms, *p* = 0.016, *n* = 13). Between baseline and 48 weeks EoT significant reduction in liver fat (5.17% vs 2.65%, *p* = 0.027) and an increase in reported LIC (0.913 vs 0.950 mg/g, *p* = 0.021) was observed.

**Conclusion:**

Liver cT_1_ decreases in patients with chronic HCV undergoing successful DAA treatment. The relatively fast reduction in cT_1_ suggests a reduction in inflammation rather than regression of fibrosis.

**Electronic supplementary material:**

The online version of this article (10.1007/s00261-020-02860-5) contains supplementary material, which is available to authorized users.

## Introduction

Chronic hepatitis C virus (HCV) affects an estimated 71 million people worldwide [1] and is associated with the development of liver cirrhosis, hepatocellular carcinoma (HCC), liver failure and death. Direct-acting antiviral therapies (DAAs) have high rates of viral eradication (> 95%) and have transformed the treatment of HCV [2–12].

Currently, even after achieving sustained virological response (SVR), patients with HCV liver cirrhosis are recommended for HCC surveillance by ultrasound combined with alpha-fetoprotein (AFP) every 6 months indefinitely [13]. However, the risk factors for development of HCC post SVR are not well defined. Recent evidence suggests that sustained virological response (SVR) reduces the risk HCC, albeit not removing the risk completely [14–16]. Fibrosis-4 (FIB-4) score > 3.25 and models involving age, platelet count, serum aspartate aminotransferase/alanine aminotransferase (AST/ALT) ratio and albumin have been identified as predictors of HCC post SVR [17, 18].

There is a growing body of evidence showing a reduction in non-invasive biomarkers of fibrosis in significant proportions of patients following DAA treatment [19–26]. However, it is unclear whether these changes are due to regression of inflammation or fibrosis, or both. In a study of paired liver biopsy pre and post DAA therapy, the maintenance or regression of fibrosis and inflammation varied across patients, while non-invasive markers overestimated histological regression of fibrosis [27]. Furthermore, it is unclear whether changes in fibroinflammation reduce risk of HCC development.

Liver fibrosis and inflammation increases the liver’s extracellular water content, which can be quantified by the magnetic resonance imaging (MRI) parameter T_1_, when corrected for iron content [28] (cT_1_). Liver cT_1_ correlates with liver fibrosis, inflammation and ballooning [29, 30], has excellent repeatability and reproducibility [31] and can predict clinical outcomes [32]. In the same scan T_2_* can quantify liver iron content (LIC [33]) and proton magnetic resonance spectroscopy (1H-MRS) can quantify liver fat content, having been validated extensively against histology [34]. Liver cT_1_ has been used to assess treatment response in patients with non-alcoholic steatohepatitis (NASH) [35] and therefore could be used to assess change in the liver parenchyma after HCV cure by DAAs.

The primary aim of this study was to evaluate changes in liver cT_1_ (fibroinflammation), T_2_* (LIC) and ^1^H-MRS (liver fat) in patients with chronic HCV following treatment with DAAs. Secondary aims were to assess changes in other non-invasive biomarkers of liver disease including transient elastography (TE), simple blood tests and serum-based fibrosis scores.

## Patients and methods

In this single center, prospective, observational cohort study patients with chronic HCV were consecutively invited from hepatology outpatient clinics at the John Radcliffe Hospital, Oxford, UK, between December 2014 and September 2017. Chronic HCV was defined by positive HCV RNA assays on more than two occasions 6 months apart. A sustained virological response (SVR) was defined as a negative HCV RNA 12 weeks post DAA treatment. All patients with a most recent clinical TE measurement > 8 kPa who were approved to start treatment with DAAs by a multi-disciplinary team were invited to take part in the study. Exclusion criteria were contraindications to MRI. Patients were treated with the DAA treatment regimens prescribed by their clinical care team, which were not affected by participation in the study. Patients underwent study assessments comprising MRI, TE and blood sampling at baseline (before treatment), and a 24 weeks and 48 weeks after the end of treatment (EoT). The study was approved by the local Research Ethics Committee (ref no: 13/SC/0234) and conforms to the declaration of Helsinki. All patients gave written, informed consent to take part.

### MRI protocol

All MRI scans were carried out at the University of Oxford Centre for Clinical Magnetic Resonance Research (OCMR) on a 3T Siemens Tim Trio scanner (Erlangen, Germany). Patients underwent the Liver*MultiScan*™ (Perspectum Diagnostics Ltd, Oxford, UK) acquisition protocol for T_1_ and T_2_* mapping and ^1^H-MRS, after fasting for at least 4 h, as described previously [28, 29].

### MRI analysis

T_1_ and T_2_* maps were analyzed using Liver*MultiScan*™ software (Perspectum Ltd, Oxford UK) for the purpose of generating and analyzing cT_1_ maps by the following steps:

1. Three regions of interest (ROIs) were placed manually on T_2_* color maps to obtain a representative LIC (estimated in units of mg/g dry weight).
2. This LIC was fed into an algorithm that transforms the measured T_1_ map into a cT_1_ map in such a way that corrects for any underestimation of T_1_ caused by increased LIC [28].
3. On the cT_1_ map, the liver was segmented semi-automatically, excluding the blood vessels, large bile ducts and other abnormalities (e.g., cysts). The mean value of cT_1_ within the liver was calculated and taken as the final cT_1_ value.

In the derivation of T_1_ and T_2_* maps, R^2^ maps are generated, which are a measure of the goodness of fit of the MR signal intensity to T_1_ and T_2_* relaxation curves. When placing ROIs in the T_1_ and T_2_* maps, mean *R*^2^ values were also calculated. T_2_* map *R*^2^ values had to be > 0.95, and T_1_ map R^2^ values had to be > 0.99 for all liver measurements to be considered valid.

^1^H-MRS acquisitions were combined and fitted using the OXSA toolbox [36] implementation of the AMARES algorithm [37] with an in-house MATLAB (The Mathworks, Natick, MA, USA) script [38]. Liver fat was expressed as a ratio of the total fat signal divided by the total water + fat signal (%).

### Transient elastography

TE (Fibroscan®, Echosens, Paris, France) was performed by a trained professional, and a valid reading was defined according to the manufacturer’s criteria (10 valid shots and IQR/median ratio < 0.3). TE was performed on the same day as the MRI scan and the blood tests where possible, alternatively the most recent clinically performed value was used (maximum 1 weeks’ interval).

### Blood sampling

Blood samples were drawn on the same day as the MRI scan. Simple scores for serum-based fibrosis markers FIB-4 [39], aspartate aminotransferase to platelet ratio index (APRI) [40] and AST/ALT ratio were calculated.

### Clinical data and anthropometric measures

Diabetes status, alcohol consumption, evidence of decompensation, height, weight, and body mass index (BMI) measurements were recorded. Patients consuming more than 14units of alcohol per week were classed as consuming alcohol above the recommended limits. Patients with cirrhosis were defined by Ishak fibrosis stage ≥ F5 on a clinically indicated biopsy or having clinical decompensation at baseline assessment.

### Patient follow-up

Patients were also followed up for the development/recurrence of HCC and new liver decompensation events (ascites, variceal bleeding, hepatic encephalopathy, liver transplantation and liver-related mortality) from recruitment until the last study visit of the last patient in the study by review of their electronic patient records.

### Statistical analysis

Median and interquartile ranges (IQR) were reported for patient characteristics due to the small numbers involved. The primary outcome was the change in liver cT_1_ following DAA treatment. Secondary outcomes included changes in liver fat, LIC, liver stiffness (measured by TE), FIB-4, APRI and AST/ALT scores and other blood-based biomarkers. Paired comparisons of these parameters between study timepoints was performed using the non-parametric paired Wilcoxon test.

Temporal changes in liver cT_1_ were compared by Wilcoxon rank test between patients with and without cirrhosis. Correlations between liver cT_1_ and TE and the influence of liver fat on liver cT_1_ [41] were investigated using the Spearman’s rank correlation coefficient. We did not correct for multiple comparisons as there were small numbers of patients, this being a proof-of-principle study. Significance level for all statistical tests was set at 0.05. All statistical analysis was performed, and all plots were generated using R statistical software [42].

## Results

**Fig. 1:**
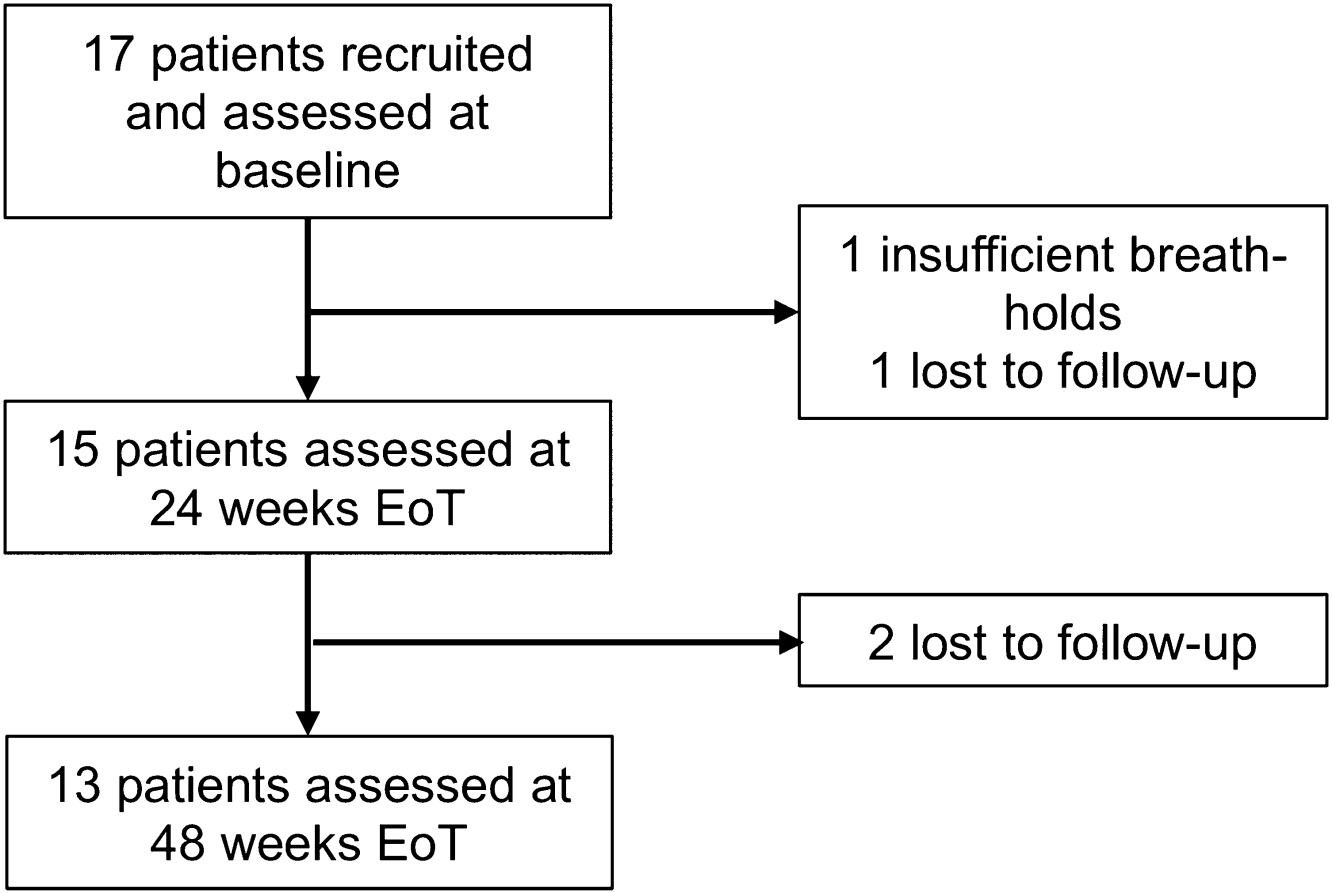
Study flow diagram. *EoT* end of treatment

Seventeen patients were recruited and assessed at baseline. One patient was excluded due to their inability to hold their breath during the MRI scan and one patient was lost to follow-up after their baseline scan. Fifteen patients returned for follow-up visits at 24 weeks EoT and were included in analysis, of which 2 were lost to follow-up at 48 weeks EoT (Fig. 1). All patients achieved SVR as confirmed by undetectable HCV RNA assay 24 weeks after treatment.

### Patient characteristics

The fifteen patients included in the analysis had median (IQR) age 58 (52–60), median (IQR) BMI 25.7 (23.2–28.4) kg/m^2^ and 10 (75%) were male. Seven (47%) patients had genotype 1 (GT1) infection, three (20%) had GT2 and five (33%) had GT3. Four (27%) patients self-reported alcohol consumption exceeding the recommended limits and seven (47%) had cirrhosis at baseline. Individual patients’ details are reported in Supplementary Table 1.

### Liver cT_1_

**Fig. 2:**
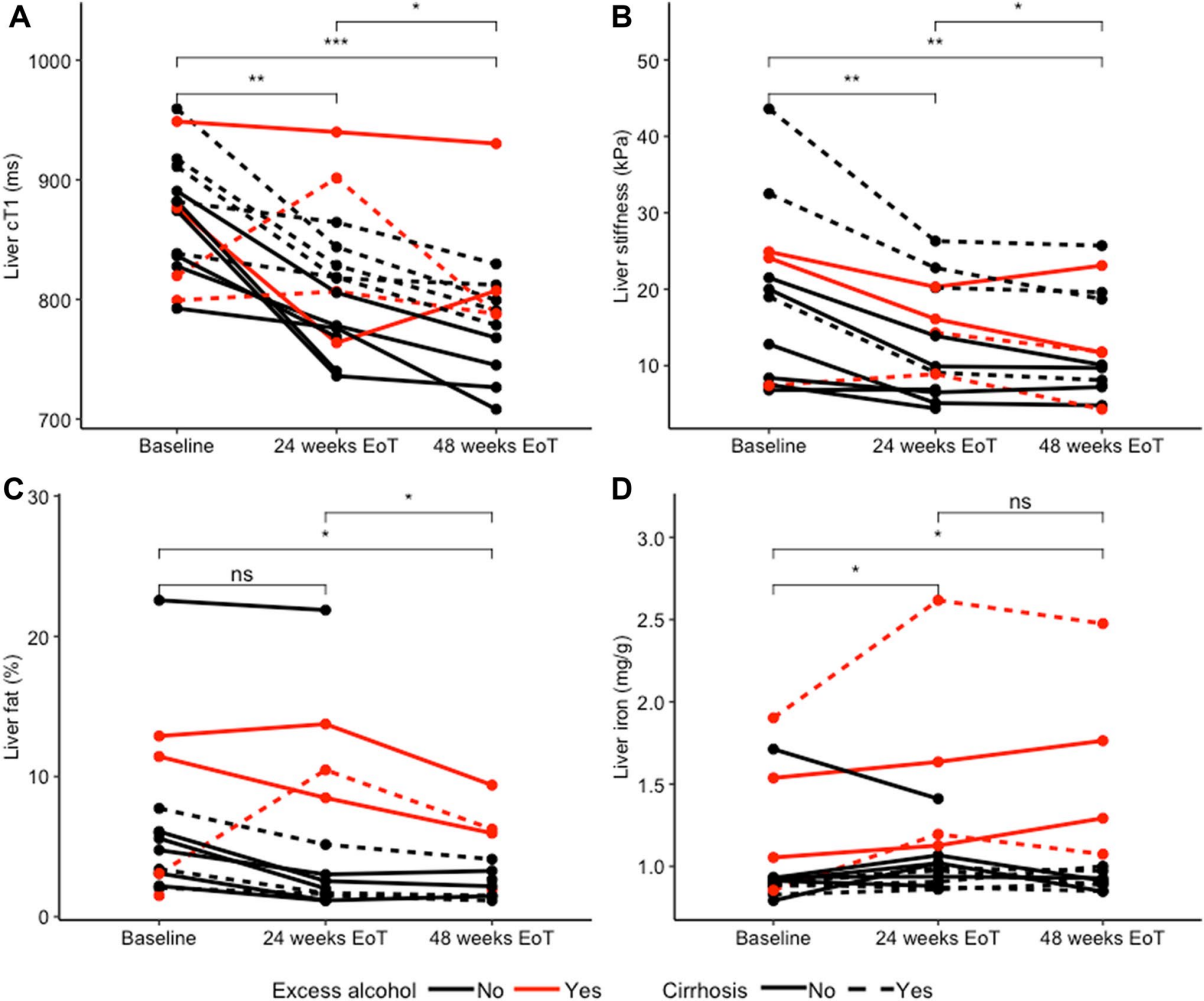
Response in non-invasive markers of liver disease to DAA treatment from baseline to 24 weeks EoT, and 48 weeks EoT. Plots show response to treatment by **a** liver cT_1_, **b** transient elastography (TE), **c** liver fat, **d** liver iron content. Each line represents an individual patient. * denotes *p* < 0.05, ** denotes *p* < 0.01, ns denotes *p* > 0.05. *EoT* end of treatment, *DAA* direct-acting antiviral therapy

**Fig. 3:**
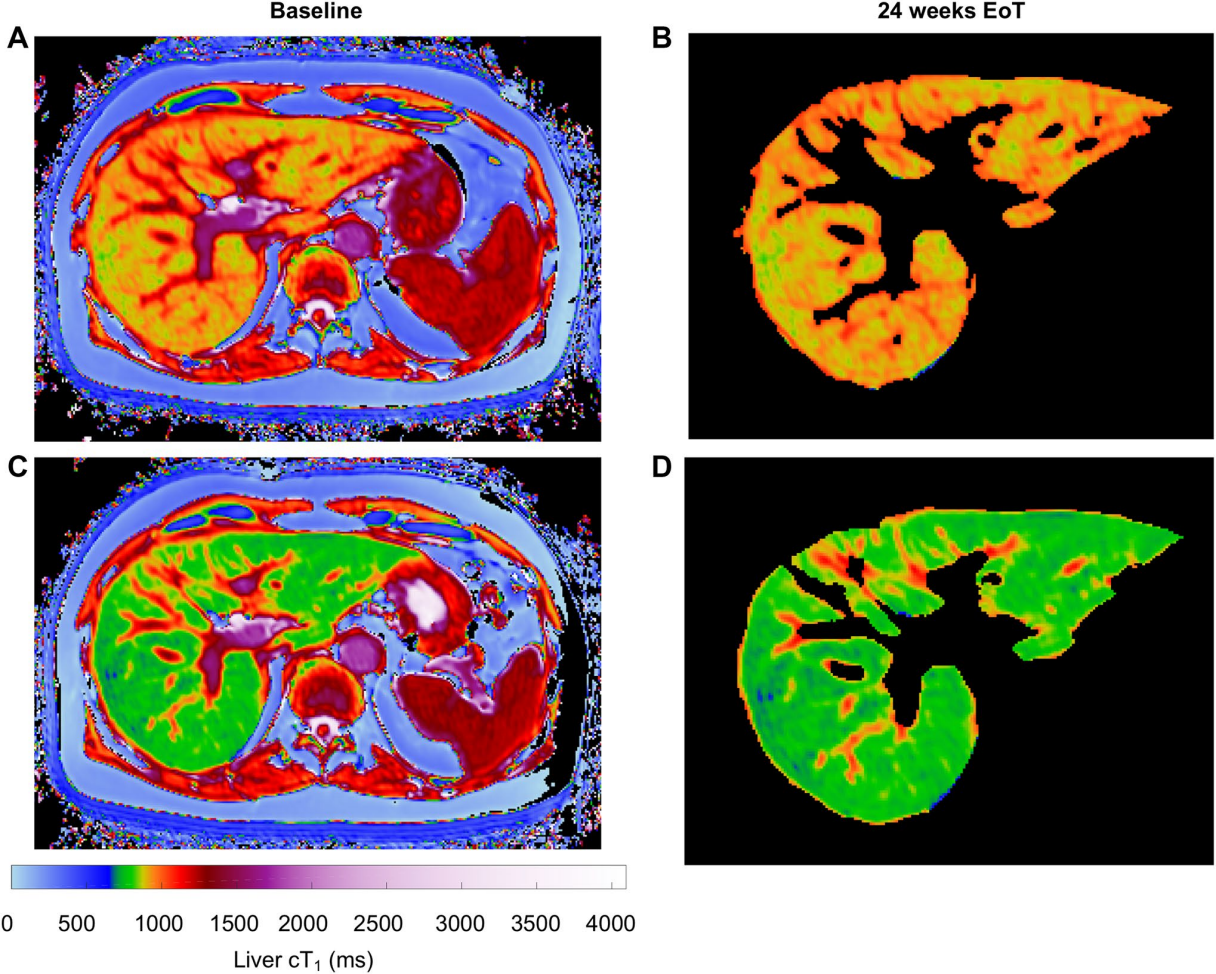
Abdominal cT_1_ images and segmentation masks pre and post treatment. Abdominal cT_1_ images are shown from a patient with chronic hepatitis C virus at baseline (**a**) and at 24 weeks after end of treatment (EoT) (**b**), together with the semi-automatically segmented liver cT_1_ masks from the same patient at baseline (**c**) and 24 weeks EoT (**d**). Mean liver cT_1_ at baseline was 874 ms and 24 weeks EoT was 735 ms

Liver cT_1_ showed a significant decrease from baseline to 24 weeks EoT (876 vs 806 ms, *p* = 0.002, *n* = 15, Fig. 2a), from baseline to 48 weeks EoT (876 vs 788 ms, *p* = 0.0002, *n* = 13) and from 24 weeks EoT to 48 weeks EoT (806 vs 788 ms, *p* = 0.016, *n* = 13, Table 1, Fig. 2a). Example images at baseline and 24 weeks EoT are shown in Fig. 3.

**Table 1:** Patient demographics at baseline, 24 and 48 weeks after the endo of treatment (EoT)

	Baseline (*n* = *15*) median (IQR)	24 weeks EoT (*n* = *15*) median (IQR)	48 weeks EoT (*n* = *13*) median (IQR)	*p* value (baseline vs 48 weeks)
BMI (kg/m^2^)	25.7 (23.2–28.4)	26.4 (23.8–27.5)	26.6 (24.3–30.7)	
Bilirubin (mmol/L)	12 (8–16)	12 (10–18)	9 (9–13)	0.0658
ALT (IU/L)	120 (59–134)	28 (21–34)	23 (17–29)	** < 0.001**
ALP (IU/L)	96 (70–152)	71 (60–105)	77 (64–93)	**0.0137**
Albumin (g/L)	36 (34–37)	39 (37–40)	38 (36–39)	**0.0172**
GGT (IU/L)	107 (88–135)	36 (24–71)	33 (24–73)	** < 0.001**
AST (IU/L)	94 (67–140)	32 (29–42)	29 (25–35)	**0.0078**
Ferritin (µg/l)	184 (42–531)	57 (10–169)	92 (57–184)	0.156
Transferrin saturation (%)	35 (17–43)	31 (17–44)	33 (26–40)	0.751
Trigs (mmol/L)	0.99 (0.70–1.64)	0.78 (0.62–1.00)	0.70 (0.61–0.97)	0.0840
HDL (mmol/L)	1.1 (0.9–1.4)	1.4 (1.2–1.6)	1.5 (1.1–1.6)	**0.0453**
LDL (mmol/L)	2.5 (2.2–2.8)	2.5 (2.3–2.9)	2.5 (2.3–2.7)	0.8885
Total:HDL ratio	4.22 (3.07–5.00)	2.90 (2.60–4.03)	2.94 (2.71–3.63)	**0.0098**
Platelets (× 10^9^/L)	192 (126–239)	223 (127–233)	233 (113–286)	0.0661
Non-invasive scores of liver disease severity				
Child–Pugh score	6 (5–6)	5 (5–5)	5 (5–5)	0.3458
FIB-4	2.51 (1.96–4.48)	2.27 (1.57–3.73)	1.39 (1.16–4.06)	**0.0156**
AST/ALT	1.158 (0.703–1.196)	1.400 (1.160–1.467)	1.29 (1.052–1.771)	0.0781
APRI	1.185 (0.776–2.299)	0.406 (0.259–0.541)	0.219 (0.181–0.658)	**0.0156**
TE (kPa)	19.5 (8.2–24.3)	11.9 (7.4–19.2)	10.1 (7.4–18.7)	**0.0020**

*ALT* alanine aminotransferase, *ALP* alkaline phosphatase, *AST* aspartate aminotransferase, *GGT* gamma-glutamyl transferase, *Trigs* triglycerides, *HDL* high density lipoprotein, *LDL* low density lipoproteins, *FIB-4* fibrosis-4, *APRI* AST to platelet ratio index, *FIB-4* fibrosis – 4, *AST/ALT score* aspartate aminotransferase/alanine aminotransferase ratio, *APRI* aspartate aminotransferase to platelet ratio index, *TE* transient elastography

### Liver fat

Liver fat measurements were available for 12 patients at baseline and 24 weeks EoT and in 11 patients at 24 and 48 weeks EoT. Median liver fat showed a trend towards decrease from baseline to 24 weeks EoT (5.17% vs 2.54%, *p* = 0.064, *n* = 12) reaching a significant decrease between baseline and 48 weeks EoT (5.17% vs 2.65%, *p* = 0.027, *n* = 11). There was no correlation between change in liver fat and change in cT_1_.

### Liver iron

LIC increased between baseline and 24 weeks EoT (0.913 vs 1.019 mg/g dry weight, *p* = 0.022, *n* = 15) and between baseline and 48 weeks EoT (0.913 vs 0.950 mg/g, *p* = 0.021, *n* = 13), but there was no significant change between 24 and 48 weeks EoT (*p* = 0.470, Table 1, Fig. 2d).

### Transient elastography

TE showed significant decreases between baseline and 24 weeks EoT (19.5 vs 11.9 kPa, *p* = 0.005, *n* = 12), between baseline and 48 weeks EoT (19.5 vs 10.1 kPa, *p* = 0.005, *n* = 10) and between 24 and 48 weeks EoT (11.9 vs 10.1 kPa, *p* = 0.041, *n* = 12, Table 2, Fig. 2b).

**Table 2:** Magnetic resonance parameters at baseline, and 24 and 48 weeks after the end of treatment (EoT)

	Baseline (*n* = *15*) median (IQR)	24 weeks EoT (*n* = *15*) median (IQR)	48 weeks EoT (*n* = *13*) median (IQR)	*p* value (baseline vs 48 weeks)
Liver cT_1_ (ms)	876 (832–901)	806 (772–836)	788 (767–807)	** < 0.001**
Liver fat (%)	5.17 (2.64–6.03)	2.54 (1.63–8.48)	2.65 (1.71–5.03)	**0.027**
Liver iron (mg/g)	0.913 (0.884–0.994)	1.019 (0.922–1.161)	0.950 (0.895–1.129)	**0.021**

TE measurements were significantly correlated with liver cT_1_ (*r* = 0.60, *p* < 0.0001, *n* = 39, Supplementary Figure S1) with a moderate correlation observed in pre-treatment measurements (*r* = 0.547, *p* = 0.05, *n* = 12) and a weaker correlation observed in post-treatment measurements (*r* = 0.134, *p* = 0.003, *n* = 27). Reductions in cT_1_ correlated moderately (*r* = 0.500, *p* = 0.015, *n* = 23) with reductions in TE.

### Blood-based biomarkers

From both baseline to 24 weeks EoT and baseline to 48 weeks EoT, significant reductions were observed in ALT (*p* < 0.001 and *p* < 0.001), AST (*p* = 0.004 and *p* = 0.008), ALP (*p* = 0.014 and *p* = 0.014), GGT (*p* < 0.001 and *p* < 0.001), FIB-4 (*p* = 0.023 and *p* = 0.016), APRI (*p* = 0.008 and *p* = 0.016), and significant increases were observed in albumin (*p* = 0.005 and *p* = 0.017) and HDL (*p* = 0.028 and *p* = 0.045).

From baseline to 24 weeks EoT significant reductions were observed in AST/ALT (*p* = 0.027) and ferritin (*p* = 0.023). From baseline to 48 weeks EoT significant reductions were observed in cholesterol:HDL ratio (*p* = 0.010).

Between 24 and 48 weeks EoT, AST, ALT, GGT and APRI showed trends towards decrease (*p* = 0.059, *n* = 11; *p* = 0.077, *n* = 12; *p* = 0.076, *n* = 13 and *p* = 0.064, *n* = 10, respectively) but no significant reductions were observed. A significant increase in platelet count was observed (*p* = 0.041, *n* = 10).

### Patient follow-up

No de novo occurrences of HCC or recurrences of HCC were observed following DAA treatment.

### Influence of cirrhosis on liver cT_1_

**Fig. 4:**
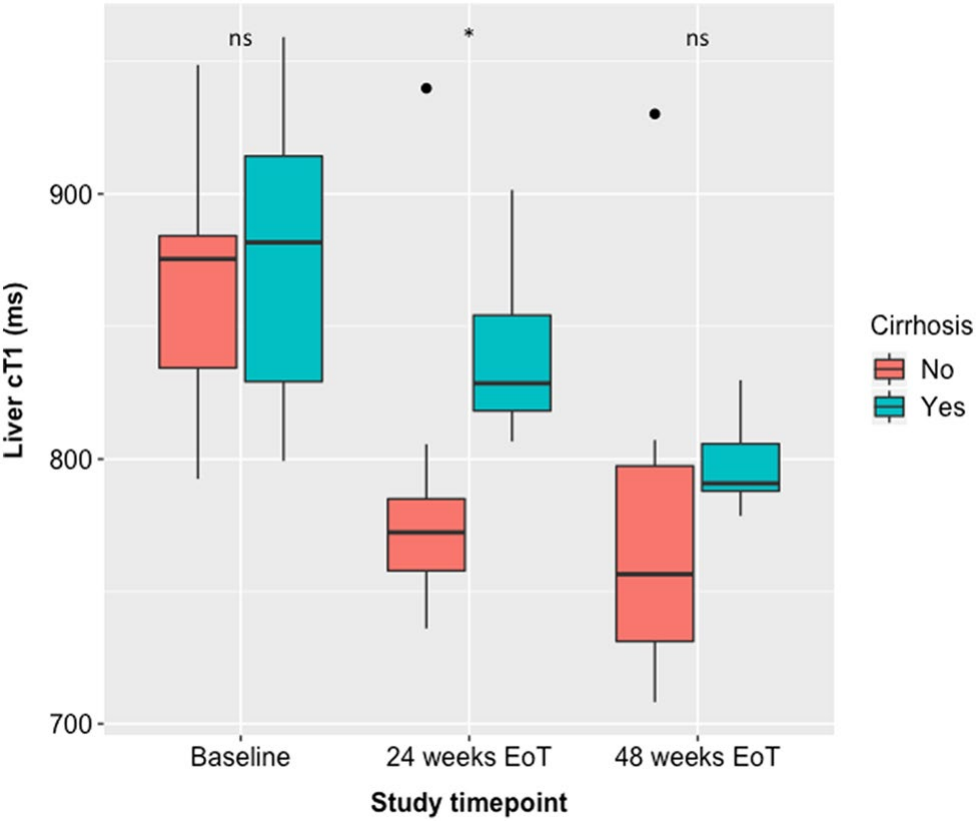
Comparison of liver cT_1_ between patients with and without cirrhosis at baseline, 24 weeks EoT and 48 weeks EoT. *EoT* end of treatment, * denotes *p* < 0.05. Dots represent outliers

Seven patients had clinical or histologically diagnosed cirrhosis. There was no significant difference in mean baseline cT_1_ between patients with and without cirrhosis (863 vs 866 ms, *p* = 0.694). Liver cT_1_ was significantly higher in the group with than without cirrhosis at 24 weeks EoT (840 ms vs 789 ms, *p* = 0.014, Fig. 4). Between baseline and 24 weeks EoT there was a significant drop in cT_1_ in patients without cirrhosis (859 vs 767 ms, *p* = 0.008) but not in patients with cirrhosis (863 vs 827 ms, *p* = 0.375). There was no significant difference in mean cT_1_ at 48 weeks between patients with and without cirrhosis (*p* = 0.234) nor any difference in magnitude of reduction in cT_1_ between 24 and 48 weeks EoT. Both groups were overall significantly reduced at 48 weeks EoT from baseline (*p* = 0.031 for both).

### Effects of alcohol consumption on non-invasive markers of liver disease

Patients with self-reported excess alcohol consumption throughout the study (*n* = 4) had significantly higher levels of LIC (1.55 mg/g vs 0.96 mg/g dry weight, *p* < 0.001), liver fat (9.19% vs 3.91%, *p* = 0.004) and GGT levels (133 vs 59 IU/L, *p* = 0.011) than those without excess alcohol consumption.

When these patients were excluded from the analysis, liver cT_1_ showed a significant decrease from baseline to 24 weeks EoT (874 vs 798 ms, *p* < 0.001, *n* = 11), from baseline to 48 weeks EoT (877 vs 773 ms, *p* = 0.004, *n* = 9) and from 24 to 48 weeks EoT (808 vs 773, *p* = 0.004, *n* = 9). Liver fat showed a significant decrease from baseline to 24 weeks EoT (6.38 vs 4.47%, *p* = 0.004, *n* = 9), from baseline to 48 weeks EoT (5.17 vs 2.53%, *p* = 0.031, *n* = 6), but not from 24 to 48 weeks EoT (*p* = 0.313). TE showed a significant decrease from baseline to 24 weeks EoT (19.1 vs 11.7 kPa, *p* = 0.008, *n* = 9), baseline to 48 weeks EoT (22.5 vs 12.0 kPa, *p* = 0.016, *n* = 7) and showed a trend towards decrease from 24 to 48 weeks EoT (*p* = 0.08, *n* = 8). LIC did not change between any timepoints when excluding those patients who consumed excess alcohol.

## Discussion

In this study, liver cT_1_ has been used for the first time to track liver fibroinflammatory response to DAA treatment of chronic HCV. The reductions in liver cT_1_ from baseline to 24 and 48 weeks EoT suggest both short and medium-term improvements in liver fibroinflammatory disease with HCV treatment.

Our results are consistent with a similar sized study by Bradley et al.(*n* = 17) at 1.5 T, in which early reductions in liver T_1_ of 35 ms were observed after DAA-achieved SVR [19]. Our reductions of 70 ms in liver cT_1_ at 3T by 24 weeks EoT were twice as much as Bradley’s result. This is to be expected as the magnitude of liver T_1_ increases (almost linearly) with magnetic field strength. This reduction in cT_1_ is clinically meaningful, being nearly two times the cT_1_ repeatability coefficient and comparable to reductions in cT_1_ observed in a study investigating response to treatment for non-alcoholic steatohepatitis (NASH) [31, 35, 43].

In our study, as the reductions in cT_1_ between 24 and 48 weeks EoT were smaller than the reductions between baseline and 24 weeks EoT, most of the reduction in cT_1_ was likely due to resolving inflammation rather than fibrosis as liver fibrosis reversal has shown to be a slow process [44]. In contrast, the reduction in liver stiffness between baseline and 24 weeks EoT, but not between 24 and 48 weeks EoT conflicts with the assumption that TE is correlated only with fibrosis, but that inflammation is significantly contributing to the TE signal.

Liver fat and cholesterol:HDL ratio reduced significantly between baseline and 48 weeks EoT. Hepatic lipid accumulation is associated with chronic HCV and steatosis has been shown to be an independent risk-factor for HCC development with chronic HCV-related cirrhosis [45]. Reduction in liver fat may therefore also improve patients’ outcomes, but a larger cohort with longer follow-up than our study would be required to investigate this. Our results suggest that HCV cure by DAA treatment may reduce liver fat regardless.

A small but statistically significant increase in LIC was observed from baseline to 48 weeks EoT. The reported LIC is a surrogate marker, calculated from and inversely proportional to the T_2_* relaxation time. T_2_* itself is a composite of the T_2_ relaxation time and magnetic field inhomogeneity. It is likely that liver tissue T_2_ has fallen (as observed by Bradley et al. [19]) with improvement in liver fibroinflammation, causing a decrease in T_2_* and increase in reported LIC in turn, rather than an increase in actual tissue iron. An increase in LIC was observed in each patient who consumed excess alcohol (Fig. 2d), but no significant change was observed in patients who did not. This indicates that the increase in LIC is driven solely by excess alcohol and suggests that when monitoring these patients following treatment, one should not assume that a small drop in T_2_* necessarily equates to an increase in tissue iron. Serum ferritin levels have been shown to fall in patients following viral eradication [46]. This could mean that after treatment the liver regains more capacity to store iron and takes up excess iron circulating in serum. Alternatively, a decrease in ferritin could be a response to reduction in inflammation, secondary to viral eradication. The latter scenario could explain our findings of increases in T_2_*-derived LIC and the observed trend towards decrease in ferritin but maintenance of transferrin saturations. Paired iron profiles were not available in all patients which may be the reason for the reduction in ferritin not reaching significance.

Patients drinking excess alcohol had higher liver fat and LIC across all timepoints in the study. Higher histological liver iron has also previously been observed in patients with both alcohol related liver disease (ArLD) and HCV than with HCV alone [47]. The reference value for elevated LIC is 1.7 mg/g [48] and our patients were at the higher end of the normal spectrum. It is known that patients with ArLD frequently present with evidence of high ferritin and/or iron overload, and even moderate alcohol consumption can increase markers of iron storage [49]. The only participants whose liver cT_1_ did not decrease between each time point were those that had been drinking heavily. Alcohol causes ongoing inflammation and fibrosis, which in continued alcohol abuse is a persistent upwards trajectory. Although HCV cure may reduce the contribution of HCV to inflammation and fibrosis, it may not be sufficient to reduce liver cT_1_ overall in these patients.

Patients without cirrhosis showed a greater initial decrease in liver cT_1_ than those with cirrhosis. We have also shown that liver cT_1_ continues to decrease between 24 and 48 weeks EoT, albeit at a lower rate than between baseline and 24 weeks EoT. Single liver cT_1_ measurements cannot separate the individual contributions of liver fibrosis and inflammation, and presently the only method that can do this is liver biopsy. However, these results indicate that relative temporal cT_1_ response could differentiate between patients with cirrhosis vs non-cirrhosis and inflammation vs fibrosis reduction. Paired biopsy would be needed to confirm this but was outside the scope of this study. It also indicates that most of the improvement in fibroinflammation in patients without cirrhosis is observed by 24 weeks EoT, which is supported by the study by Bradley et al. [19] who observed significant short-term T_1_ changes.

Multiple studies have reported significant decreases in TE measurements and in blood-based biomarkers of fibrosis in patients undergoing DAA treatment in as little as 12 weeks and up to as long as 12 months EoT using TE [20–26, 50], in accordance with our findings. We observed a weaker correlation between liver cT_1_ and TE after treatment than prior to treatment (*r* = 0.134 vs 0.547). While TE correlates well with histological liver fibrosis in untreated HCV [51–53], a study has shown it to have poorer ability to identify cirrhosis in patients post-HCV treatment than in patients pre-treatment [54]. Using a cut-off of > 12 kPa for cirrhosis in that study, 21% of patients classified without cirrhosis had cirrhosis and 38% of patients with cirrhosis post-treatment were misclassified. In our study, it is likely that a significant reduction in inflammation has affected the post-treatment correlation between liver cT_1_ and TE.

Liver cT_1_ has been shown to predict higher risk of liver-related clinical outcomes [32], including HCC. We observed no new incidence of HCC in any DAA-treated patient; however, this was a small cohort with a relatively short follow-up. Alongside fibrosis and inflammation epigenetic change may also affect HCC risk post SVR. Epigenetic aberrations are linked to tumor development. Chronic HCV has been shown to induce epigenetic changes, which persist following viral eradication by DAAs [55, 56]. It is postulated therefore that some patients, regardless of whether a reversal in either fibrosis or inflammation takes place, may still be at risk of HCC development due to persistent epigenetic alterations. Longer follow-up in larger cohorts is needed in these patients who have undergone fibroinflammatory reversal, as structural changes in the liver and residual fibroinflammation may have an influence on the prognostic value of liver cT_1_.

In addition to the small numbers and relatively short follow-up, the greatest limitation of this study is the lack of paired histology. However, liver fat measurement with MRS is well-validated against histology and liver cT_1_ has been shown to correlate well with histological fibrosis, inflammation and ballooning [29, 30]. Additionally, liver biopsy is not a true gold standard against which to measure due to sampling variation and interobserver variability [57, 58]. Furthermore, the current staging system for fibrosis may not be optimal in the context of fibrosis regression in viral hepatitis [59]. Additionally, we only investigated patients with TE > 8 kPa at baseline, whereas patients with lower baseline fibroinflammation may exhibit more varied changes in liver cT_1_.

In this study we observed reductions in liver cT_1_ and liver fat following DAA therapy. Changes in both these parameters have also been observed in response to resolution of non-alcoholic steatohepatitis (NASH) [35]. This indicates that multiparametric MRI may be of use as a monitoring biomarker.

Further studies should investigate whether multiparametric MRI also changes in response to the treatment of other liver diseases (e.g., alcohol cessation in ArLD). Long term follow-up for HCC and other liver-related complications is also needed to evaluate the prognostic capability of changes in liver cT_1_ in patients with HCV after successful DAA treatment.

In conclusion, this study has shown that liver cT_1_ decreases after DAA treatment for chronic HCV. It is unclear to what extent these reductions are influenced by inflammation or fibrosis regression but may represent inflammatory change accompanied later by concurrent fibrosis regression.

## Electronic supplementary material

Below is the link to the electronic supplementary material.

Supplementary Material 1 (DOCX 678 kb)

## Data Availability

Data are available from the corresponding author on reasonable request.
