# Supplementary material for: Liver cT1 decreases following direct-acting antiviral therapy in patients with chronic hepatitis C virus
Source: Abdom Radiol (NY). 2020 Nov 28;46(5):1947–57. doi: 10.1007/s00261-020-02860-5 (PMC8131342; doi:10.1007/s00261-020-02860-5)
Supplement: Supplementary file 1 — Supplementary Material 1 (DOCX 678 kb) [file 261_2020_2860_MOESM1_ESM.docx]

***Supplement to:***

**Liver cT_1_ decreases following direct acting antiviral therapy in patients with chronic hepatitis C virus.**

Arjun N. A. Jayaswal^1^, Christina Levick^1,2^, Jane Collier^2^, Elizabeth M. Tunnicliffe^1^, Matthew D. Kelly^4^, Stefan Neubauer^1^, Eleanor Barnes^2,3,5^¶, Michael Pavlides^1,2,3^*¶.

1. Oxford Centre for Clinical Magnetic Resonance Research, Division of Cardiovascular Medicine, Radcliffe Department of Medicine, University of Oxford, Oxford, UK.
2. Translational Gastroenterology Unit, University of Oxford, Oxford, UK
3. Oxford NIHR Biomedical Research Centre, Oxford, UK
4. Perspectum Ltd, Oxford, UK
5. Peter Medawar Building for Pathogen Research, University of Oxford, UK

¶ Joint senior Author.

* Corresponding author.

email: [michael.pavlides@cardiov.ox.ac.uk](mailto:michael.pavlides@cardiov.ox.ac.uk) .

**Tables**

Table S1 – Individual baseline patient characteristics and self-reported alcohol consumption at each study visit …… 2

**Figures**

Figure S1 – Liver cT_1_ vs transient elastography (TE) ………………………………………………………………….. 3

**Table S1: Individual baseline patient characteristics and self-reported alcohol consumption at each study visit**

| **Baseline decompensation** | | No | No | No | Yes (HCC) | No | Yes (ascites) | No | No | No | No | No | No | No | Yes (ascites) | No |
| --- | --- | --- | --- | --- | --- | --- | --- | --- | --- | --- | --- | --- | --- | --- | --- | --- |
| **Baseline cirrhosis** | | Yes | Yes | Yes | Yes | No | Yes | No | No | No | Yes | No | No | No | Yes | No |
| **Alcohol consumption (units/week)** | **48**  **weeks** | 0 | 140 | 5 | 0 | 0 | 0 | 14 | 0 | 14 | 0 | 0 | 130 | 14 | 30 | 28 |
|  | **24 weeks** | 0 | 140 | 5 | 0 | 0 | 0 | 0 | 0 | 14 | 0 | 0 | 65 | 14 | 50 | 30 |
|  | **Baseline** | 0 | 110 | 14 | 0 | 0 | 0 | 0 | 0 | 14 | 0 | 0 | 130 | 14 | 140 | 32 |
| **BMI** | | 27.1 | 18.9 | 26.8 | 23.2 | 24.8 | 27.0 | 22.6 | 24.9 | 23.9 | 32.1 | 30.3 | 28.4 | 22.9 | 25.7 | 41.5 |
| **Sex** | | M | M | F | F | M | M | M | F | M | F | M | M | F | M | M |
| **Age** | | 60 | 57 | 54 | 60 | 67 | 58 | 48 | 68 | 58 | 65 | 45 | 59 | 28 | 53 | 52 |
| **Treatment duration (weeks)** | | 24 | 12 | 12 | 12 | 12 | 12 | 12 | 12 | 8 | 8 | 12 | 12 | 12 | 16 | 12 |
| **HCV genotype** | | 3 | 1 | 1 | 1 | 2 | 3 | 1 | 1 | 3 | 2 | 3 | 2 | 1 | 1 | 3 |
| **DAA treatment used** | | Sofosbuvir, Ledipasvir + Ribavarin | Ombitasvir, Paritaprevir, Ritonavir,  Dasabuvir + Ribavarin | Sofosbuvir, Ledipasvir + Ribavarin | Sofosbuvir, Ledipasvir + Ribavarin | Sofosbuvir + Ribavarin | Daclatasvir, Sofosbuvir + Ribavarin | Ombitasvir, Paritaprevir, Ritonavir,  Dasabuvir + Ribavarin | Elbasivir, Grazoprevir | Glecaprevir, Pibrentasvir | Glecaprevir, Pibrentasvir | Glecaprevir, Pibrentasvir | Glecaprevir, Pibrentasvir | Elbasivir, Grazoprevir | Elbasivir, Grazoprevir + Ribavarin | Glecaprevir, Pibrentasvir |

**FIGURES**


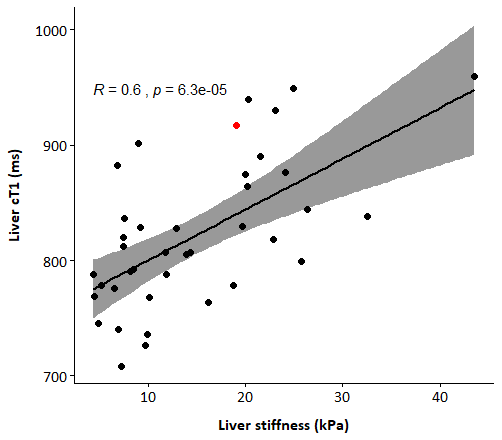


**Figure S1:** **Liver cT_1_ plotted against paired TE measurements**. Both liver cT_1_ and liver stiffness measured by transient elastography (TE) were taken on the same day, within an hour of each other, except one measurement which was taken within a week of MRI scan (coloured in red). Shaded area indicates 95% confidence interval, R denotes Spearman’s rank correlation coefficient.
